# Supplementary material for: Health and wellbeing of indigenous older adults living in the tea gardens of Bangladesh
Source: PLoS One. 2021 Mar 4;16(3):e0247957. doi: 10.1371/journal.pone.0247957 (PMC7932146; doi:10.1371/journal.pone.0247957)
Supplement: S1 Appendix — (PDF) [file pone.0247957.s001.pdf]

Operation Research on

Assessing Health Status and Quality of life of Indigenous (Teagarden Workers) Elder Population (60 years or above) at selected tea gardens in Moulvibazar district of Bangladesh

Section 1: Socio-demographic information		
**Questions**	**Write/circle the correct code**	**Skip**
101. District	☐	
102. Upazila	☐	
103. Name of tea garden	☐☐	
104. Village		
105. Area/Line/Para		
106. Name of respondent	☐☐	
107. Mobile Number of respondent	Date/Month/Year	
108. Date of interview		
109. Name of interviewee		
110. Code no. of question paper	District/Upazila/ Code of Tea Garden/Code of information collector/Respondent Code	
111. Respondent's date of birth	Date/Month/Year	
112. Age of respondent		
113. Gender of respondent	Male… 1<br>Female… 2<br>Other… 88	
114. Educational qualification of respondent		
115. Religion of respondent	Islam … 1<br>Hindu … 2<br>Buddhist … 3<br>Christian … 4<br>Other (please mention) … 88	
116. Caste/group/title of respondent	….	
117. Marital status of respondent	Married… 1<br>Unmarried/ never married… 2<br>Widow/widower … 3<br>Abandoned by husband/divorcee/ separated … 4	
118. What is your profession?	Household work… 1<br>Permanent tea worker… 2<br>Contract tea worker… 3	

	Studying… 4<br>Job… 5<br>Teaching… 6<br>Daily labour… 7<br>Other (please mention) … 88	
119. What is your weekly income?	… Taka<br>No income… 00	
120. Is your present income for meeting daily livelihood and needs?	Yes… 1<br>No…. 2	**201**
121. If not, who bears the additional expenses?	Child …1<br>Relative… 2<br>Social/Loan Centre… 3<br>Other (please mention) … 88	

Section 2: Health Seeking Behaviour		
**Questions**	**Write/circle the correct code**	**Skip**
201. Are you/have you suffered from any critical/long-term illnesses?	Yes… 1<br>No… 2	
202. If yes, for how many months? (duration)		
203. Did you see any medical test or treatment for this (or this type of) illness?	Yes… 1<br>No… 2	**208**
204. Where did you get the treatment or medical tests done?	District/Sadar Hospital… 1<br>Upazila Health Complex (UHC)… 2<br>Union Health & Family Welfare Centre (UH&FWC)… 3<br>Community Clinic… 4<br>NGO Clinic/Private Hospital/Clinic …5<br>Chamber of passed doctor… 6<br>House… 7<br>Chamber of non-pass doctor… 8<br>Hospital at tea garden… 9<br>Dispensary… 10<br>Pharmacy… 11<br>Ayurvedic/homeopathic chamber… 12<br>Other (please mention)… 88	
205. Who performed your medical tests or treatment? (Could be more than one doctor, write chronologically?	Care-giver<br>Order<br>Passed doctor… 1<br>Medical Assistant/ SACMO… 2<br>Nurse… 3<br>Health Assistant (HA)/ Family Welfare Assistant (FWA)… 4	Reason

	Paramedic … 5<br>Village midwife… 6<br>Non-pass doctor… 7<br>Midwife… 8<br>Compounder… 9<br>Dresser… 10<br>Volunteer… 11<br>Ayurvedic/homeopathic doctor … 12<br>Pharmacist… 13<br>Kabiraj… 14<br>Other (please mention)… 88	
206. Was there any delay between symptoms of illness and getting health care?	Yes… 1<br>No… 2	**209**
207. What was the duration of delay between symptoms of illness and getting health care?	Hours/minutes	
208. Why was there a delay/did you not want medical care?	Unavailable to afford treatment expense/poverty<br>Did not have money… 1<br>Did not know seriousness of illness… 2<br>Did not have someone to take me … 3<br>Did not have right to receive treatment … 4<br>Did not think it was important… 5<br>Did not know where to go… 6<br>Did not go due to far distance… 7<br>Did not have transport to go… 8<br>Did not have time to go… 9<br>Family did not allow… 10<br>Did not have someone to look after my child… 11<br>It was not possible to go at the time the health centre was open… 12<br>It was not possible to go due late hours/disaster or crime risk… 13<br>Quality of care not good… 14<br>Behaviour of care giver not good… 15<br>No one available at health centre… 16<br>Other (please mention)… 88	
209. Were you under regular check-up?	Yes … 1<br>No… 2	**211**

210. Did you take the prescribed medicines correctly?	Yes… 1<br>No… 2	**211**
211. If no, why did you not take prescribed medicines correctly?	Unavailable to afford treatment expense/poverty<br>Did not have money… 1<br>Did not know seriousness of illness… 2<br>Did not have someone to take me … 3<br>Did not have right to receive treatment … 4<br>Did not think it was important… 5<br>Did not know where to go… 6<br>Did not go due to far distance… 7<br>Did not have transport to go… 8<br>Did not have time to go… 9<br>Family did not allow… 10<br>Did not have someone to look after my child… 11<br>It was not possible to go at the time the health centre was open… 12<br>It was not possible to go due late hours/disaster or crime risk… 13<br>Quality of care not good… 14<br>Behaviour of care giver not good… 15<br>No one available at health centre… 16<br>Other (please mention)… 88	
212. Did you ever receive elder population health care?	Yes… 1<br>No… 2	

Section 3: Assessment of Health Condition			
**Questions**	**Write/circle the correct code (Please tick)**		
301. What are the long-term illnesses you are suffering from? (could be more than one answer):	**Critical**	**Not Critical**	**Normal**
Vision… 1<br>Hearing… 2<br>Mobility… 3<br>Mental… 4<br>Nutrition… 5<br>Long-term coughing/respiratory… 6<br>Blood pressure… 7<br>Diabetes… 8<br>Gastric… 9			

Reproductive (Ovary, Fistula) … 10<br>Kidney/Urinary Tract… 11<br>Cancer… 12<br>Heart related… 13<br>Other (please mention)… 88			
302. Impact of daily physical activities	**Limited**	**Very Limited**	**High**
Agriculture, carrying water etc. type of difficult work… 1<br>Moving things around the house etc. type of medium intense work… 2<br>Carrying container etc. type of light work… 3<br>Climbing mountains, hills, stairs… 4<br>Climbing mountains, hills, stairs more than once… 5<br>Bending back/knees/lowering head … 6<br>Walking one yard… 7<br>Walking more than a yard… 8<br>Walking one mile or more… 9<br>Other (please mention) … 88			

Section-4: Instrument for assessing the quality of life of indigenous older adults from the tea gardens of Bangladesh.

Questions	Response Values				
	1	2	3	4	5
My physical ability does not limit my daily activities	Strongly Disagree	Disagree	Somewhat Agree	Agree	Strongly Agree
I have freedom to make my own decision and able to do things I like	Strongly Disagree	Disagree	Somewhat Agree	Agree	Strongly Agree
I'm not too worried with how I'm going to die	Strongly Disagree	Disagree	Somewhat Agree	Agree	Strongly Agree
I’m satisfied with the scope and opportunities of life	Strongly Disagree	Disagree	Somewhat Agree	Agree	Strongly Agree
I’m satisfied with my level of social activity	Strongly Disagree	Disagree	Somewhat Agree	Agree	Strongly Agree
I have good relationship with my family, friends, and neighbours	Strongly Disagree	Disagree	Somewhat Agree	Agree	Strongly Agree
