# Supplementary material for: Health and wellbeing of indigenous older adults living in the tea gardens of Bangladesh
Source: PLoS One. 2021 Mar 4;16(3):e0247957. doi: 10.1371/journal.pone.0247957 (PMC7932146; doi:10.1371/journal.pone.0247957)
Supplement: S2 Appendix — (PDF) [file pone.0247957.s002.pdf]

Assessing Health Status and Quality of life of Indigenous (Teagarden Workers) Elder Population (60 years or above) at selected tea gardens in Moulvibazar district of Bangladesh

Guideline for In-depth Interview (IDI) conduction

Target Group:
HCPs (Doctors, Health Supervisors, Compounders, Midwives) in the dispensaries of teagardens

Guideline:

Overview of health status of older population at tea gardens

- Please tell us about your opinion or idea about the health of the older (60 or above) population at the tea garden
- What are the illnesses that the older population of the tea garden generally suffer from?
- Which age group generally suffers from this type of illnesses? Who suffers the most in terms of gender (male/female), age, religion, caste and why?
- Why do you think they suffer from these illnesses?

The healthcare-seeking behaviour of older population at tea gardens

- Where do the older population at tea gardens go to for medical tests or treatment?
- What kind of medical facilities are available at your hospital for the older population at tea gardens?
- Please tell us about the quality of treatment, availability of sufficient quantity of medicines and location of receiving healthcare, particularly for older people
- Please tell us about the facilities available in this hospital based on the referrals made by the older population

Challenges in receiving health care by the older population at tea gardens

- What kind of challenges (financial/social/familial/ignorance) do the older population at tea gardens face for receiving healthcare?
- What are the problems they face while receiving healthcare at hospitals?
- How do you think these problems can be solved?

Advice on improving health care system for the older population at tea gardens

- How do you think the use of health services by local older population for their health conditions can be increased?
- What kind of initiatives will increase the quality of service provided by your health care centre for the older indigenous adults?
- What can be done to increase the quality of service and referral management for the elder population at tea gardens?
