# Supplementary material for: Health and wellbeing of indigenous older adults living in the tea gardens of Bangladesh
Source: PLoS One. 2021 Mar 4;16(3):e0247957. doi: 10.1371/journal.pone.0247957 (PMC7932146; doi:10.1371/journal.pone.0247957)
Supplement: Attachment — Submitted filename: PLOs one review.docx [file pone.0247957.s004.docx]

The paper is very informative on factors that hinder the health status and quality of life of indigenous people in Bangladesh. Many different issues have been raised in this study and of value for institutions and practitioners that deal with the improvement of living conditions, health care, and quality of life of indigenous people of tea gardens in Bangladesh.

The Introduction is offering a well-given overview of the problems of indigenous people in Bangladesh while Discussion is providing a critical review of the findings. The main disadvantages of this manuscript are related to text structure for which recommendations for improvement have been given. The following sections offer suggestions for improvement of this paper:

ABSTRACT

- The stated aim of the study that is, "This study explores the health status and quality of life of indigenous older people in Bangladesh to help fill a gap due to the lack of knowledge and evidence on this topic" is not sufficient. Besides saying that goal of the study was looking into the health status and quality of life of indigenous people it would be good to mention that its determinants were investigated also.

INTRODUCTION

- page 5, line 96 "…covering health, education, the economy, and political rights (8)(9)." Suggesting to enclose both reference numbers together within brackets ("…covering health, education, the economy, and political rights (8, 9).").
- page 5 line 105 Suggesting to put both reference numbers together in brackets e.i."(11, 12)"
- page 5 line 108 Suggesting to enclose both reference numbers together in brackets
- page 6 line 117 Suggesting to enclose all reference numbers together in brackets
- page 6 line 120 In the last paragraph of Introduction (starting with the sentence " In Bangladesh, the few studies that did involve..") there are several sentences that attempt to provide a reader with aim of this study. Those are: "This indicates a comprehensive look is essential for exploring the health status, health needs, and their impact on the quality of life of older adults, which is still unknown and unexplored","This study, therefore, explores the health status, health seeking behaviour, and quality of life of the indigenous communities residing in the tea gardens of Bangladesh" see provide a reader with aim of study but it seems that none actually provides sufficient explanation" or " This study, therefore, explores the health status, health-seeking behaviour, and quality of life of the indigenous communities residing in the tea gardens of Bangladesh". However, I would suggest writing just one clear sentence about what the aim of the study was. Additionally, besides saying that aim of the research was looking into the health status and quality of life of indigenous people residing in tea gardens of Bangladesh only, it seems valid to add (considering analyses used in the research) that goal of the study was also defining its determinants.
- page 6 line 121 In the sentence " This indicates a comprehensive look is essential for exploring the health status, health needs, and their impact on the quality of life of older adults, which is still unknown and unexplored." authors are talking in terms of impact between variables, however, this study only tested correlations.

METHODOLOGY

- page 7 line 137 Suggesting to change current subheading (Sample size and sampling technique) into following one: "Sample and sampling technique" considering later given prepositions.
- page 7 139 Reference numbers 23 to 26 to be enclosed together in brackets
- page 7 line 140 Suggesting that both reference numbers are enclosed together in brackets
- page 8 table 1-Suggesting to exclude table 1 from the manuscript altogether because of the text before which describes the procedure, sampling, and subsample sizes in detail. There is no need to cover the same information twice.
- page 8 line 162 Study design and data collection section do not offer a detailed description of instruments used. For that reason, the following is suggested:

a) The questionnaire for measuring health status should be described in a detailed fashion i.e. how many questions the questionnaire contains, what was measured by it, how were responses organized, how was composite result calculated, etc.

b) More information should be given on measure for detecting the presence of

chronic illness (were open ended or closed questions used, how were results calculated or equivalent).

c) And, it would be good to provide the reliability that is, Cronbach's alpha for quality of life measure.

- page 9 table 2 Suggesting to exclude table 2 from this section altogether and place in Appendix (while referring a reader that it can be found there).
- page 10 line 180 Although authors explained that IDI covers questions about health status and health-seeking behaviors it would be good to list used questions in the Appendix.
- page 10 line 199 Suggesting to exclude heading "Quantitative findings" from the manuscript altogether. There is no need to indicate to a reader which category of findings will follow. Just start the Results section with the heading "Socio-demographic characteristics".
- page 11 line 201 Suggesting that after the sentence "The socio-demographic characteristics of the respondents are presented in Table-3 and Fig-1." a reader is presented with table 3 and figure 1 while associated comments are placed below them.
- page 12 line 221 Suggesting to add "(N=400) " after "Description of socio-demographic and health condition variables used in analysis with their percentage in total." In that way, there would be no need for notes below table 3.
- page 12 line 200 to 209 It is proposed that text under the heading "Socio-demographic characteristic of subjects" along with table 3 and figure 1 are moved to the "Sample and sampling technique" section. In that way, a description of subjects can be found in one place in the manuscript.
- page 13 line 230 After the sentence " A majority of respondents (80%) were suffering from some type of chronic disease. Fig-2" it seems appropriate to introduce a reader with figure 2 while all related comments should be found underneath it.
- page 13 line 242 Suggesting that after a sentence "Furthermore, Fig-3 presents the distribution of multimorbidities among the indigenous older adults across male and female" the reader is presented with Figure 3 while its associated comments go below the figure.
- page 14 line 251 In the sentence" Furthermore, these chronic conditions and multi-morbidities have a substantial effect on the daily lives of the indigenous older adults with a large number (69.6%) of respondents reporting difficulty in carrying out household tasks." authors are claiming that health status of subjects have an effect on their daily lives. Considering that no analysis undertaken in this study confirms this relationship it can only be stated that study results support that: "A large number (69.6%) of respondents reporting difficulty in carrying out household tasks" and which can be included in the manuscript instead.
- page 14 line 253 After referring a reader to Figure 4 by stating: " Fig-4 presents the severity of the limitation on various daily activities of indigenous older adults") it seems appropriate to present it before commenting on it.
- page 14 line 264 Considering that text which starts with a sentence: "More than one-third of respondents did not receive any treatment.." refers to figure 5 it is suggested to present figure to a reader priory commenting on it.
- page 15 line 278 Delete colon at the end of this heading
- page 15 line 279 For purpose of improving structure and comprehension of the text starting with the sentence " The multiple logistic regression shown in Table 4 reveals…" it is suggested that this section begins by stating what was tested by logistic regression, what were its predictors and a criterion variable while defining its categories (in that way, a note under the table 4 can be removed). Once this being said, table 4 should be introduced while comments associated with it would follow.
- page 16 line 296 Heading of table 4 is insufficient. To provide a reader with a more informative heading I suggest putting the following heading for table 4: "The results of multiple logistic regression analysis on predicting persons suffering from chronic disease based on age, gender, marital status, income, and education."
- page 17 line 301 It seems good to introduce a reader to table 5 straight after a sentence: "Furthermore, Multiple linear regression was run to predict 301 quality of life from age, gender,income, education, marital status, and condition of chronic disease" while comments related to it would be given below the table.
- page 17 line 302 Before commenting on the results of analysis (that is, before a sentence: "Age and status of chronic conditions were significantly associated with quality of life."), it would be good to provide an explanation what is the difference between the unadjusted and adjusted model of the analysis to understand findings with greater clarity. Also, it would be informative to explain how independent variables were coded before the presentation of results in the table.
- page 18 line 323 Heading of table 5 is not sufficient. To be more informative heading should enclose that table 5 shows results of unadjusted and adjusted models of linear regression analysis predicting quality of life of indigenous people by age, gender, marital status, income education, literacy, and presence of chronic disease.
- page 18 Table 5 is not comprehensive enough. The column with variable names is too narrow. Try to fit variable names in one line. Also, if an explanation of how independent variables were coded for this analysis is provided before the table, variable names could be narrowed to marital status, income, education, chronic disease.
- page 19 line 331 Suggesting that heading itself ("qualitative findings") is removed from the manuscript
- page 19 line 332 Suggesting that after a sentence " The healthcare workers of tea garden health facilities have shared their views and recommendations on the health status and health-seeking behavior of the indigenous older population, along with its associated challenges" authors add one more sentence providing the reader with a list of themes which emerged from qualitative analysis.
- page 21 line 377-388 Citation: " When the older people face any injury; The 377 y ask for X-ray and believe that they will be fine after performing it. They considered X-ray as a treatment for injury. I try to convince them, but it is very difficult to change their believe. That’s how much ignorant they are about their medical conditions”- (IDI-5, Teagarden hospital)"is placed in the text after covering alcohol issues of subjects however, considering that the given example describes the ignorance of indigenous people, it seems better to put this quote after following sentence "The health-seeking behaviours of the older population living in tea gardens was considered very poor by all the respondents, with ignorance and lack of awareness being the main identified cause." at page 20 line 370.
- page 22 line 409 It is unusual to have abbreviation in the heading so it is suggested to remove it from here and introduce abbreviation in the first sentence under this heading i.e. " An awareness-raising programme among older people in the tea gardens that focuses on health education and personal hygiene was felt to be essential by healthcare providers (HCPs) that took part in the interviews for this study."
- page 22 line 409 It is suggested to add "within tea gardens" to this heading to unify all the subheadings in this section i.e. "Recommendations of Healthcare Providers on the healthcare of older people within tea gardens".

DISCUSSION

- page 24 line 470 A sentence "These conditions have a substantial impact on daily life activities too with 70% of indigenous older people reporting difficulties with performing household tasks." implies there is causation between health conditions of subjects and their experience of difficulties with household tasks which haven't been tested in this study. Therefore, this sentence needs to be reformulated in a way that its author's assumption that health conditions and disruption of daily tasks are related.
- page 25 line 487 It is suggested to enclose all reference numbers together in brackets
- page 27 line 527 It is suggested to enclose all reference numbers together in brackets
- page 27 line 539 It is suggested to enclose all reference numbers together in brackets
- page 27 line 539 Based on study findings authors suggest ( in a sentence "This study can serve as an evidence base for further in-depth research and policy interventions.") that certain policy interventions could be implemented for indigenous people in Bangladesh although, none are mentioned. Therefore, it is suggested that a couple of words are shared regarding the practical implications of this study.

FIGURE 5

- The explanation for abbreviations used in Figure 5 is missing in order all information provided in it is tangible.
