# Supplementary material for: Health and wellbeing of indigenous older adults living in the tea gardens of Bangladesh
Source: PLoS One. 2021 Mar 4;16(3):e0247957. doi: 10.1371/journal.pone.0247957 (PMC7932146; doi:10.1371/journal.pone.0247957)
Supplement: Attachment — Submitted filename: Response to Reviewers_PLOSone.docx [file pone.0247957.s005.docx]

**Response to Reviewers**

I am grateful to both reviewers for their constructive reviews and helpful recommendations. I tried to address all the proposed revisions to the best of my capability. The detail response to the suggested revisions are given below:

**Text structural revisions**

All the suggested structural revisions by both reviewer-1 and reviewer-2 have been addressed. These include:

- Putting more than one references enclosed together in a single bracket.
- Exclusion of Table 1 and Table 2.
- Exclusion of headings like: ‘Quantitative findings’ and ‘Qualitative findings’.
- Placing all Tables and Figures first when mentioned and then placing the associated description/comments below them.
- Revising headings (delete colon, delete abbreviation)
- Change of headings for Table 4 and 5. (the headings have been changed as suggested)
- Completing references, (Ref no: 1,3,6,7,8,9,10,13,32).

**Response to content revisions**

**Response to Reviewer-1**

| **Section** | **Page and Line** | **Comment** | **Response** |
| --- | --- | --- | --- |
| **Abstract** | Page 2, Line: 25-27 | ‘Besides saying that goal of the study was looking into the health status and quality of life of indigenous people it would be good to mention that its determinants were investigated also.’ | The aim of the study is amended as follows:  ‘This study explores the health status and quality of life along with their determinants among indigenous older people in Bangladesh in order to fill the knowledge and evidence gap on this topic.’ |
| **Introduction** |  |  |  |
|  | Page 6 line 120 | Suggestion to write one clear sentence stating the aim of the study with mentioning about determinants. | It has been amended as follows:  ‘This study, therefore, aims to explore the health status and quality of life and to define its determinants among the indigenous older adults residing in tea gardens of Bangladesh.’ |
|  | Page 6 line 121 | Authors are talking in terms of impact between variables, however, this study only tested correlations. | This line has been amended as follows:  “‘However, the healthcare needs, health status, and its relationship with quality of life of indigenous older adults in Bangladesh are still unknown and unexplored, which signifies the need for research specific to this community.’’ |
| **Methodology** | Page 7 line 137 | Suggesting to change current subheading (Sample size and sampling technique) into following one: "Sample and sampling technique" | Subheading has been amended to “Sample and sampling technique” |
|  | Page 8 line 162 | ‘Study design and data collection section do not offer a detailed description of instruments used.’ Some suggestions were also offered to make this section more comprehensive. | A separate section for instrument has been added with detail description of the questions used and their measurement technique. All the suggestions have been addressed. Can be found at Page-10, line-193 of the new manuscript.  The questionnaire has also been attached as Appendix-1. |
|  |  | ‘It would be good to provide the reliability that is, Cronbach's alpha for quality of life measure.’ | Cronbach's alpha value has been added-  The Cronbach's alpha of the quality of life measurement scale was 0.66. |
|  | page 10 line 180 | Suggested to add IDI guidelines in Appendix. | The guideline used for conducting IDI has been added in Appendix-2. |
| **Results** | page 12 line 200 to 209 | It is proposed that text under the heading "Socio-demographic characteristic of subjects" along with table 3 and figure 1 are moved to the "Sample and sampling technique" section. | Table-3 (now Table 1) and Fig 1 along with their associated texts have been moved to the "Sample and sampling technique" section. |
|  | Page 14 line 251 | Suggestion to amend the line based on analysis of the manuscript. | The line has been amended as per suggestion to-  "A large number (69.6%) of respondents reported difficulty in carrying out household tasks" |
|  | Page 19 line 332 | Suggestion to add one more sentence providing the reader with a list of themes which emerged from qualitative analysis. | The comment has been addressed as follows- ‘Three broad themes were emerged from the qualitative analysis- health scenario of the older adults, health-seeking behavior of the older adults, challenges faced by older adults in maintaining good health.’ |
|  | page 15 line 279 | ‘it is suggested that this section begins by stating what was tested by logistic regression, what were its predictors and a criterion variable while defining its categories’ | A para has been added in response describing the variables used in logistic regression and what was measured by them. This can be found at Page 15 line 318 to 323 of the new/revised manuscript. |
|  | page 17 line 302 | It was suggested that before explaining multiple linear regression result – ‘it would be informative to explain how independent variables were coded before the presentation of results in the table.’ | A para has been added in response explaining the coding of the independent variables. It can be found at page 17 line 349 to 357 of the new manuscript. |
|  | page 18 Table 5 | Table 5 is not comprehensive enough. The column with variable names is too narrow. Try to fit variable names in one line. | Table 5 (now Table 3) has been amended as suggested. Can be found at page 18 Table 3. |
|  | page 19 line 332 | ‘add one more sentence providing the reader with a list of themes which emerged from qualitative analysis.’ | It has been amended as follows: ‘The themes emerged from the qualitative analysis were common health problem of the indigenous older adults, practice of preventive health behavior, factors associated with health status and health-seeking behavior, inequality in health condition, challenges of older people in maintaining good health, and recommendation for improving health condition and health services to indigenous older adults.’ |
|  | page 21 line 377-388 Citation | Changing the citation under page 20 line 370 | Amended as suggested. |
|  | page 22 line 409 | It is suggested to add "within tea gardens" to this heading to unify all the subheadings in this section’’ | Amended as suggested. |
| **Discussion** | Page 24 line 470 | Suggestion to reformulate the sentence- "These conditions have a substantial impact on daily life activities too with 70% of indigenous older people reporting difficulties with performing household tasks." | The sentence has been reformulated as follows-  “Almost all (90%) the respondents were suffering from any kind of chronic health problem and a large number (70%) of them reported of having difficulties with performing household tasks. This indicates a possibility that the chronic condition might have an influence in limiting their daily activities, which can be investigated with further studies.” |
|  | Page 27 line 539 | It is suggested that a couple of words are shared regarding the practical implications of this study. | It has been addressed by adding the following sentence-  “This study implies the need for policies to improve the coverage of the old-age welfare system, including marginalized groups such as indigenous peoples, and to develop tailored health funding and awareness-raising strategies for indigenous older adults.  health strategies for older population etc.” |
|  | Figure-5 | The explanation for abbreviations used in Figure 5 is missing in order all information provided in it is tangible. | Full form of the abbreviations have been added. |

**Response to Reviewer 2:**

**Comment 1:**

Quantitative data’s results, the presentation of the results of the instrument for assessing the quality of life used are not clear, specifically with regard to the relationship between the analysis the answers and the other sample variables.

**Response:**

To increase the clarity of the instrument and analysis for assessing quality of life, a description of multiple linear regression analysis exploring the relationship between quality of life and predictor variables (socio-demographic & chronic condition) is added into the result section before the table, explaining how the variables were coded and a clarification of adjusted and unadjusted models. The Cronbach alpha score of the quality of life score scale is also added.

**Comment 2:**

Qualitative data’s results, it remained to include the questions asked in the ten-in-depth interviews, as well how was the triangulation process of categorization (which steps were followed?). Methodologically it should also be referred the minimum and maximum duration (time) of the ten-in-depth interviews, and it should be given more than just one respondent’s answers to justify each categories found.
To mention that the research meets all applicable standards for the ethics of experimentation and research integrity.

**Response:**

The IDI guideline has been attached as Appendix 2.

Couple of lines have been added mentioning data triangulation and duration of the interviews- IDI participants were selected from health service providers of different educational and social background as part of the data triangulation process. The respondent were 3 registered medical doctors, 3 compounders, 3 paramedics, and one community healthcare worker. Each interview lasted from 30 to 60 minutes. Can be found at page 9 line 184-188 of new manuscript.

Another line has been added mentioning triangulation of qualitative and quantitative findings at page-12 line 252-254. ‘The qualitative data were used to supplement the quantitative findings and triangulated interpretations were discussed to demonstrate a comprehensive health scenario of the indigenous older adults.’

Ethics statement has been added to the methodology section- ‘Informed written consent was taken from all the respondents. Anonymity and confidentiality of the respondents were maintained throughout the data collection, handling, and analysis process. Ethical approval was taken from Institutional Ethical Review Board of CIPRB.’ (page 10 line 189-191)
